# Supplementary material for: Interventions promoting recovery from depression for patients transitioning from outpatient mental health services to primary care: Protocol for a scoping review
Source: PLoS One. 2023 Sep 15;18(9):e0291559. doi: 10.1371/journal.pone.0291559 (PMC10503712; doi:10.1371/journal.pone.0291559)
Supplement: S1 Appendix — (PDF) [file pone.0291559.s001.pdf]

S1 Appendix

List of abbreviations/concepts

Abbreviation / concept	Definition
MDD	Major Depressive Disorder
DSM-IV	The Diagnostic and Statistical Manual of Mental Disorders, Fifth Edition
ICD-10	International Classification of Diseases and Related Health Problems 10th Revision
Co-design	A participatory approach to design interventions in collaboration with stakeholders.
Stakeholder	Any individual or group who is responsible for or affected by health- and healthcare-related decisions that can be informed by research evidence [82]. In this study, stakeholders are patients, general practitioners, psychiatrists, nurses, job consultants, medical social workers, and researchers.
Patients transitioning	When patients move between care settings. In this study, we focus on patients' transition from outpatient mental health services to primary care.
